# Research on Tire Marking Point Completeness Evaluation Based on K-Means Clustering Image Segmentation

**DOI:** 10.3390/s20174687

**Published:** 2020-08-19

**Authors:** Yuan Yu, Jinsheng Ren, Qi Zhang, Weimin Yang, Zhiwei Jiao

**Affiliations:** College of Mechanical and Electrical Engineering, Beijing University of Chemical Technology, Beijing 100029, China; yuyuanjd@263.net (Y.Y.); renjs630@163.com (J.R.); zhangq0618@163.com (Q.Z.); yangwm@mail.buct.edu.cn (W.Y.)

**Keywords:** machine vision, tire marking point, completeness, image segmentation

## Abstract

The tire marking points of dynamic balance and uniformity play a crucial guiding role in tire installation. Incomplete marking points block the recognition of tire marking points, and then affect the installation of tires. It is usually necessary to evaluate the marking point completeness during the quality inspection of finished tires. In order to meet the high-precision requirements of the evaluation of tire marking point completeness in the smart factories, the K-means clustering algorithm is introduced to segment the image of marking points in this paper. The pixels within the contour of the marking point are weighted to calculate the marking point completeness on the basis of the image segmentation. The completeness is rated and evaluated by completeness calculation. The experimental results show that the accuracy of the marking point completeness ratings is 95%, and the accuracy of the marking point evaluations is 99%. The proposed method has an important guiding significance of practice to evaluate the tire marking point completeness during the tire quality inspection based on machine vision.

## 1. Introduction

In recent years, with the improvement of people’s living standards and the rapid development of the automobile industry, the popularity of automobile has increased significantly. More attention has been paid to the safety and comfort of the automobile. It is strict on stability and silence during high-speed driving [1]. The tires are the important parts of the automobile, their dynamic balance and uniformity are two factors affecting safety and comfort during driving [2]. In the detection of finished tires, marking points with different colors and shapes are used to mark the position information of the dynamic balance and uniformity test on tires for the counterweight during the installation of tires and their accessories, so as to improve the balance of the car with high speed [3,4]. The poor printing during the process of printing or scratching of the tire with the conveying equipment during the process of conveying will lead to incomplete marking points. Since every marking point is an important reference point for the tire installation, a less complete marking point will make it difficult for the tire installer to recognize the color, shape, and position of the marking points, which affect the accuracy of the tire and wheel hub installation. Therefore, the accuracy of the marking points should be detected before the tire is finished. In addition, it is necessary to check the marking point completeness, which is also regarded as a criterion for whether the marking points are qualified. The traditional detection of tire marking points completeness is mainly through manual subjective evaluation of the tires moving on the conveyor line, and the marking points that are judged incomplete need to be reprinted. The manual detection of the marking point completeness with high work intensity is prone to visual fatigue. In addition, because there is no objective judgment basis for completeness, misjudgment is easy to occur, and the accuracy and efficiency of the detection of marking points cannot be guaranteed.

In contrast, intelligent detection technology based on machine vision technology has been widely studied and applied in many fields [5,6,7]. Collecting images by industrial cameras, recognizing the colors and shapes of tire marking points, and calculating their completeness are effective methods for the quantitative analysis of marking point completeness. It can avoid misjudgment caused by manual subjective factors and improve the accuracy and efficiency of the marking point completeness evaluation. In various fields, there are great progresses in the study of completeness. Chu Qichao and his teammates used machine vision technology to judge the completeness of the dry package quickly and accurately on the packaging line [8]. He Chao used the method of deep learning object classification to realize online automatic germ completeness calculation during germinated rice processing [9]. In the development of the tire marking points recognition and detection system, domestic and foreign researchers focus on color and shape recognition. For example, the Quality Control System IRIS-M [10] invented by the German SICK-Sensor Intelligence company and the closed-loop control system identity CONTROL TMI 8303.I [11] invented by the German Micro–Epsilon company can perform online tire marking point recognition. The Japanese Bridgestone Corporation proposed a device to realize the printing and recognition of tire marking points [12]. The Portuguese scholar named André P. Dias used the background subtraction method to realize tire positioning [13]. Wang Yong proposed to use supporting directional machine to recognize the color of the tire marking point [14]. It is demonstrated that although some achievements of the tire marking points recognition system based on machine vision have been made, there are few reports on the calculation and evaluation of the completeness marking point after the recognition of the tire marking points. In this paper, machine vision technology is used to calculate the marking point completeness quantitatively on the basis of the color and shape recognition of the tire marking point, and then according to the calculated value of completeness, the marking point is rated and evaluated to achieve the online automatic detection of the tire marking point completeness. The method proposed in this paper blazes new trails on the marking point completeness rating, which meets the strict requirements of smart factories for the recognition and detection of the tire marking point, laying the foundation for the intelligent detection technology of the tire industry.

## 2. Completeness Calculation Scheme Based on Image Segmentation

### 2.1. Image Acquisition of the Tire Marking Point

**Figure 1:**
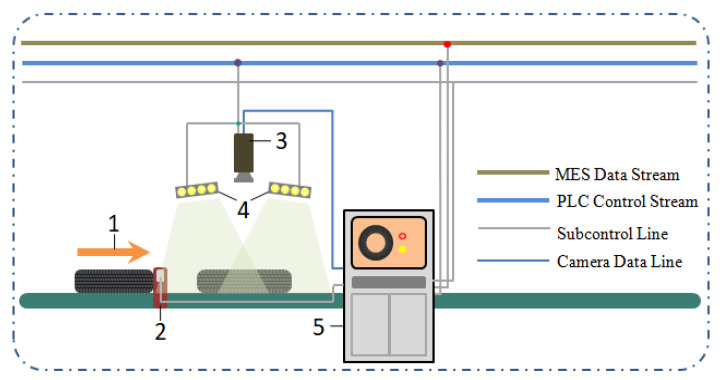
Online image collection system of the tire marking point: **1**—Direction of the tire movement, **2**—Photoelectric switch, **3**—Industrial camera, **4**—Light Emitting Diode (LED) light, and **5**—Host computer.

The online collection system of the tire the marking point’s image is shown in Figure 1. The tire on the conveyor line triggers the light source and camera according to the preset delay by the photoelectric switch sensor. When the tire passes directly under the color Charge Coupled Device (CCD) industrial camera, the host computer controls the light source to turn on and trigger the industrial camera to collect the tire image marked with the marking points and send the image to the host computer to process it. After locating and extracting the marking points, the images of the tire marking point with the size of 48 × 48 pixels can be gotten. The color and shape of the tire marking points can be recognized, and their completeness is calculated and evaluated. After that, the recognized result is transferred from the host computer to the Manufacturing Execution System (MES) and compared with the dynamic balance and uniformity detection results in the MES to determine whether the marking point is qualified. The detected result is sent to a Programmable Logic Controller (PLC) to shunt the tire, that is, the unqualified tire will be diverted into the repair stage, and the qualified tire will be sent to test by an X-ray, and then classified and stored according to a tire category. The configuration of the host computer is the Central Processing Unit (CPU) is Intel (R) Core (TM) i5-3470M; the main frequency is 3.2 GHz; the memory is 8 G; the operating system is 64-bit Windows10, and the experimental software platform is Visual Studio 2015 and OpenCV3.2.0.

**Figure 2:**
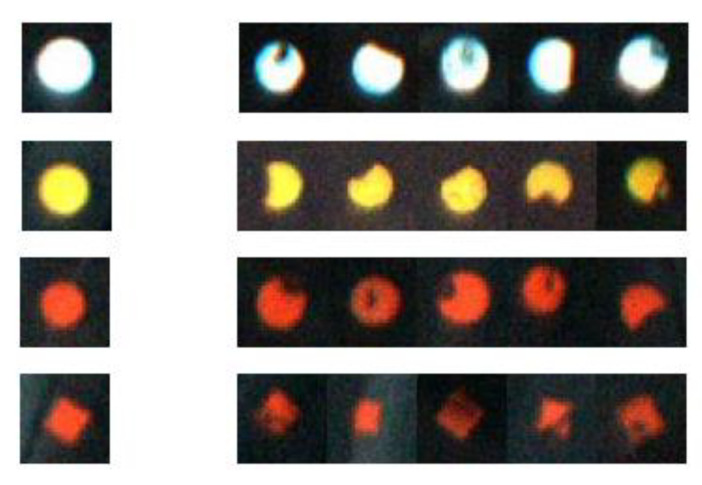
Comparisons of complete and incomplete tire marking points.

There are three colors of the tire marking points obtained by the online image collection system, including red, yellow, and white, and there are four shapes, including a solid circle, hollow circle, square, and triangle. During the process of printing the marking points, due to the poor printing, the scratches between the tires and the conveying equipment, printing on the uneven texture on the side of tires, or printing on the fur on tires, there would be incomplete marking points. The comparisons between the complete and the incomplete tire marking points are shown in Figure 2.

### 2.2. Determination of the Marking Point Segmentation Scheme

**Figure 3:**
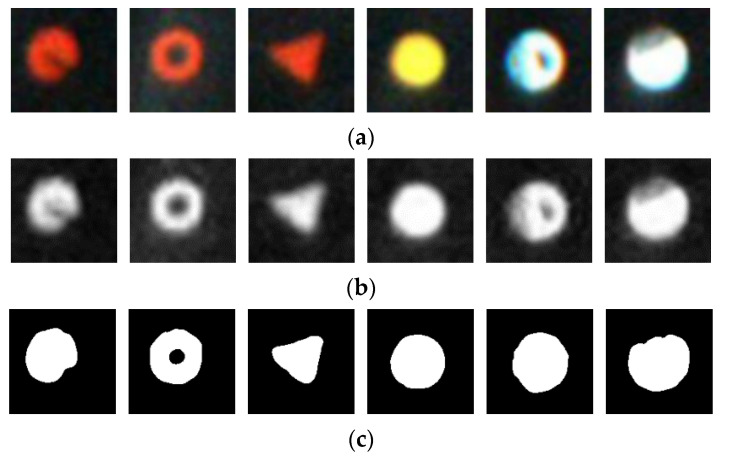
Calculation process of the marking point completeness: (**a**) The marking point image, (**b**) The gray marking point image, and (**c**) The marking point image segmented using the Otsu method.

The calculation of the marking point completeness should be divided into three steps: segmenting the marking points, extracting the contour of the complete marking point, and calculating the marking point completeness. The aim of segmenting the marking point is to separate the marking point from the background area and is a prerequisite for the calculation of the marking point completeness. The results of the segmentation directly affect the accuracy of the subsequent calculation of completeness. After extracting the contour of the complete marking point, it is a relatively simple method of calculating the completeness to use the ratio of pixels of the segmented marking point image and those of the corresponding complete marking point. The segmentation algorithms based on the edge contour or threshold are widely used, and the foreground image and the background area can be divided into two parts. One of the most popular threshold-based segmentation algorithms, is the maximum between-class variance method, also known as the Otsu segmentation method [15]. It is a global adaptive threshold segmentation algorithm. It searches for the optimal threshold based on the principle of the smallest variance within the target area or the background area and the largest variance between different areas and divides the image into a target area and a background area. Taking the Otsu segmentation method as an example to segment the marking points, shown in Figure 3. Since the marking point image is a Red-Green-Blue (RGB) three-channel image shown in Figure 3a, when it is segmented using the maximum between-class variance method, the RGB mark point image should be converted into a single-channel image first, and the conversion result is shown in Figure 3b. Then, the single-channel image is segmented by the maximum between-class variance method. The white area is the area where the marked points are obtained, and the black area is the segmented background area, shown in Figure 3c. The color is the main feature of the marking point. However, the color of the marking point image is not fully utilized, when the image is converted from the RGB three-channel one into a gray one. The maximum between-class variance method strictly divides the gray image of the marking point into the object and background by calculating the global adaptive threshold. According to the segmentation results, for example, Figure 3a,c, the blurred transition edges are segmented into the marking point foreground for the solid red circle marking point and the white solid circle marking point. It can be seen that the calculation result of the marking point completeness relies on the segmentation of the marking point absolutely. When the marking point is relatively blurred and the edge is not clear, the segmentation algorithm is based on the edge or based on the threshold value, which when used to divide the image into foreground and background, may lead to reflect the true marking point completeness unsuccessfully, shown in Figure 3.

In order to consider the edge transition between the marking point and the background, and reflect the influence of the edge area on the marked point completeness, a completeness calculation method is proposed based on weighting and clustering image segmentation. The purpose of image segmentation is to divide different characteristic regions of the image. The purpose of the clustering is to divide the data points with the same attributes into a category. Their essences are the same. The clustering method is used to divide the pixels in the image into multiple discretes so that the pixels have a high degree of similarity in each region, and there is a high comparison among these regions in order to reach the purpose of the image segmentation.

## 3. Segmentation of the Marking Point

### 3.1. Preprocess of the Marking Point

**Figure 4:**
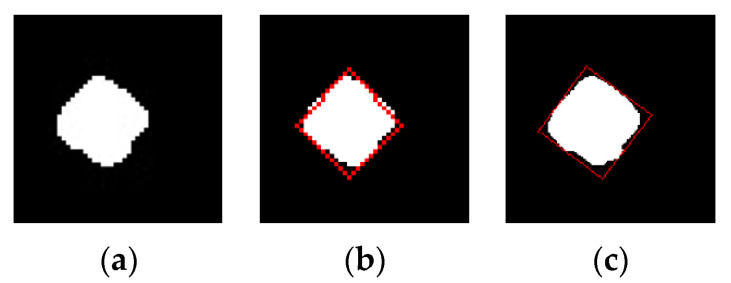
The contour of a square marking point before and after enlarging: (**a**) Segmented image of a marking point before enlarging, (**b**) Integrity contour of a marking point before enlarging, and (**c**) Integrity contour of a marking point after enlarging.

**Figure 5:**
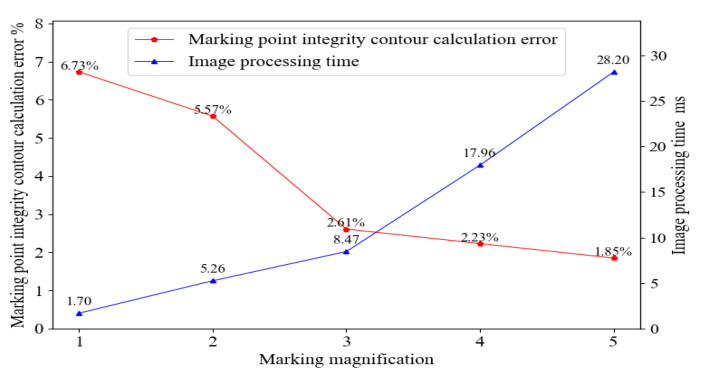
The relationship between the image magnification of the marking point and the calculation error of the integrity contour and the image processing time.

The image collection system of the tire marking point collects the tire image and captures the marking points. The image size of the obtained marking point is 48 × 48 pixels, where the marking point accounts for about 1/3–1/4 of the entire image. The low resolution of the marking point will lead to obvious jagged patterns after the marking point image is segmented, shown in Figure 4a. Moreover, in the process of extracting the contour of the complete marking point, the contour parameters of the complete marking point are calculated as real numbers, and a large error will occur in the process of transforming these real numbers into integers, which will affect the accuracy of the completeness, shown in Figure 4b. In order to make the calculation results of the marking point completeness accurate, the image is enlarged before segmentation. As shown in Figure 4c, when the image is enlarged, the error is obviously reduced, which is generated during the conversion from the contour parameter of the complete marking point to integers, and the image processing time will also increase. The experimental results show that when the magnification is greater than three times, the calculating error is not decreased significantly, from 2.61% to 1.85%, but the image processing time is increased significantly, from 8.47 millisecond (ms) to 28.20 ms, as shown in Figure 5. In a word, the image of the tire marking point is enlarged by three times, so that the speed of processing the image and the accuracy of calculating the completeness can be taken into account.

### 3.2. Image Segmentation of the Marking Point Based on the K-Means Clustering

The main clustering algorithms for image segmentation include K-means clustering [16], Fuzzy C-means (FCM) clustering [17,18], and so on. In this paper, the K-means and FCM clustering are both tested for their segmentation effect and processing speed. Although the image segmentation method of the FCM clustering has high segmenting accuracy, it needs a large amount of calculation and slow processing speed. For each marking point image, the average processing speed is 9.27 ms using K-means clustering and the average processing speed is 600.36 ms using FCM clustering, which is not suitable for the real-time requirements of the online marking point recognition system. By contrast, the K-means clustering image segmentation algorithm is simple, efficient, and with low complexity. In this paper, the K-means clustering image segmentation algorithm is used to segment the marking point.

The basic process of the K-means clustering algorithm is as follows: the *k* of the number of clusters is given, and *k* data points are selected randomly from the data set as the initial centroid *c*^0^*_j_*, *j* = 1, 2 … *k*. Equation (1) is used to calculate the distance between the remaining data points of the data set to the initial centroid and divide it into the cluster closest to the initial centroid;

(1) $$C_{j}^{t}=\{f\left(i\right):\|f\left(i\right)-{c^{t-1}}_{j}{\|}^{2}\leq \|f\left(i\right)-c_{p}{\|}^{2}:\forall p=1:k,p\neq j\},$$

where, *t* is the number of iterations, *C^t^_j_*, *j* = 1, 2, …, *k*; is the *j* cluster generated by the *t* iteration, and *f*(*i*) represents the eigenvalue of data points.

Then, the *k* clusters (*C*^1^*_j_*, *j* = 1, 2, …, *k*) are obtained. Equation (2) is used to calculate the centroid *c*^1^*_j_* of the *k* clusters *C*^1^*_j_*, and the data points in the data set are re-divided into the cluster closest to the *c*^1^*_j_* and form cluster *C*^2^*_j_*;

(2) $$c_{j}^{t}=\frac{1}{\left|C_{j}^{t}\right|}\sum_{f\left(i\right)\in C_{j}^{t}}f\left(i\right),j=1,\ldots ,k,$$

where, |*C^t^_j_*| is the number of data points contained in the *j* cluster, and Σ*f(i*) is the sum of the eigenvalues of the data points in the *j* cluster.

It is the process of cyclically iterating the distribution of the data points in the data set and updating the centroid of the cluster. When the distance from the data point in the cluster to the centroid is the smallest, it satisfies Equation (3), or the set number of clustering iterations is reached, the clustering center *c^t^_j_* and the class *C^t^_j_* of the data points in the data set to be segmented are output, and the clustering progress is ended.

(3) $$SSE=argmin_{c}\sum_{j=1}^{k}\sum_{f\left(i\right)\in C_{j}}d^{2}\left(f\left(i\right),c_{j}\right)=argmin_{c}\sum_{j=1}^{k}\sum_{f\left(i\right)\in C_{j}}\|f\left(i\right)-c_{j}\|{}^{2},$$

where, *SSE* represents the sum of squares of the Euclidean distance between the data points and the centroid of the cluster, *d*(*f*(*i*), *c_j_*) represents the Euclidean distance from the data points to the centroid, and *f*(*i*) represents the eigenvalue of the data points.

**Figure 6:**
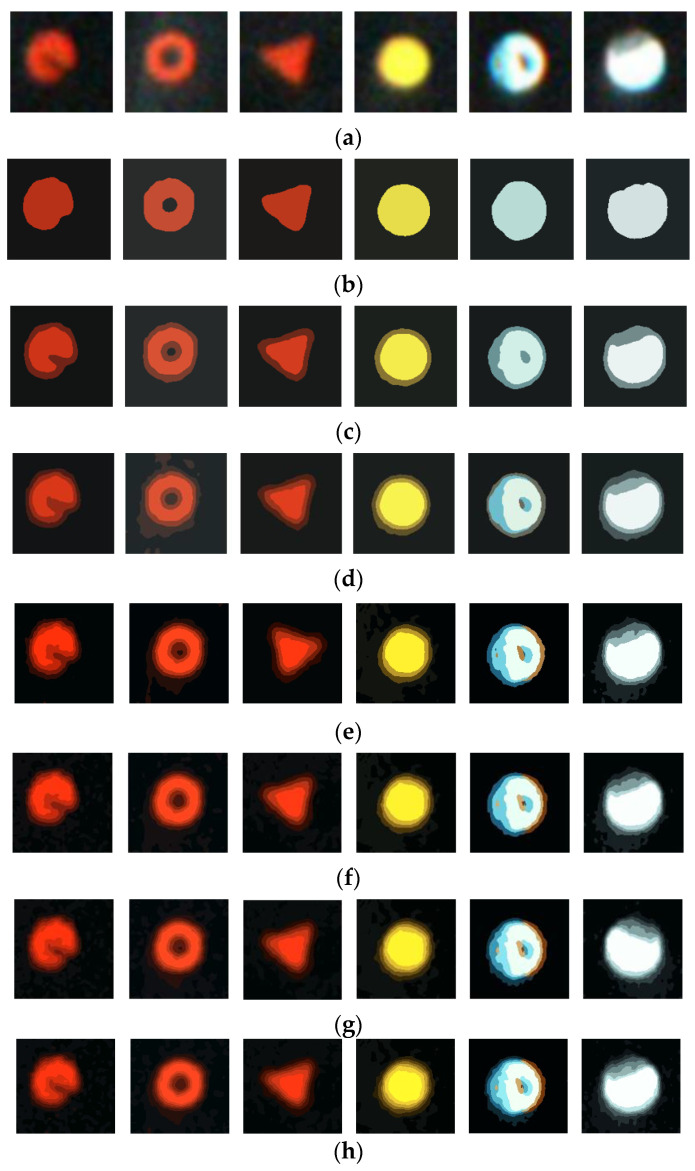
The results of the K-means clustering segmentation algorithm: (**a**) Original image of marking points, (**b**) The segmented images by clustering when *k* = 2, (**c**) The segmented images by clustering when *k* = 3, and (**d**) The segmented images by clustering when *k* = 4, (**e**) The segmented images by clustering when *k* = 5, (**f**) The segmented images by clustering when *k* = 6, (**g**) The segmented images by clustering when *k* = 7, (**h**) The segmented images by clustering when *k* = 8.

When using the algorithm, the number *k* of clusters should be determined at first. The value of *k* directly determines the results of the clustering segmentation. The segmentation results of the tire marking points from *k* is 2 to 8, and are shown in Figure 6. It shows when *k* = 2, the image is divided into the foreground (marking point) and the background, and the blurred edges may be divided into the foreground or the background. There is no edge transition between the foreground and the background, just like using the Otsu segmentation method in Section 2.2. When *k* is 3 to 8, as shown in Figure 6, the brightest area is the area of the foreground (marking point), and the darkest area is the background. The area between the brightest and the darkest areas is divided into several regions. The larger the *k* is, the finer the cluster is. It is hard to select the suitable *k* by visual inspection.

The marking point samples with the same color and shape (for example red and circle) are selected, which can be regarded as a whole image. The K-means clustering methods with the different *k* values are used to segment this image. *N_i_* is the number of pixels in a region *i* (*i* = 1, 2, …, *k*), *T* is the total pixels of the whole image. *P_i_* is indicated by the percentage of *N_i_* to *T*, which can be calculated. For K-means clustering with *k* = 3 and *k* = 4, the area with a large value of the luminance channel (V channel) of the Hue-Saturation-Value (HSV) space is divided into several regions, and the area with a small value of the V channel is a whole region, which can be regarded as the background. For example, when *k* = 4, *P*_1_ = 0.08, *P*_2_ = 0.10, *P*_3_ = 0.08, and *P*_4_ = 0.74, their corresponding values on the V channel are 0.92, 0.31, 0.62, and 0.08. The fourth region (0.08) is dark, which is the background. It makes up 74 percent of the total area of the image. When *k* is larger than 4, the background area is subdivided. For example, when *k* = 5, *P*_1_ = 0.08, *P*_2_ = 0.08, *P*_3_ = 0.08, *P*_4_ = 0.27, and *P*_5_ = 0.50, their corresponding values of the V channel are 0.93, 0.64, 0.36, 0.12, and 0.07. The fourth and the fifth regions (0.12 and 0.07) are dark. The background makes up 77 (25 and 50) percent of the total area of the image; when *k* = 8, *P*_1_ = 0.06, *P*_2_ = 0.06, *P*_3_ = 0.05, *P*_4_ = 0.05, *P*_5_ = 0.05, *P*_6_ = 0.13, *P*_7_ = 0.29, and *P*_8_ = 0.31, their corresponding values of the V channel are 0.95, 0.58, 0.38, 0.76, 0.22, 0.14, 0.09, and 0.05. The sixth, seven, and eighth regions (0.14, 0.09 and 0.05) are dark. The background (the sixth, seven, and eighth regions) makes up 73 (13, 29, and 31) percent of the total area of the image. That is to say, the background may be divided into two or several parts when *k* is larger than 4.

The purpose of segmenting the marking point image is to consider the edge transition between the foreground (marking point) and background. In fact, the dark part has little contribution to the marking point completeness. Therefore, the dark part (background) is not necessary to be divided in detail. All of the dark parts could be regarded as the background and their weights are zero, which can also simplify the weighting process for marking point completeness calculation. Thus, in this paper, *k* = 4 is selected as the clustering category in the K-means clustering segmentation.

## 4. Marking Point Completeness Calculation

### 4.1. Extract the Contour of Complete Marking Points

After the image is segmented by K-means clustering, the area with the highest brightness is the object area of the marking point. Before calculating the marking point completeness, the information of the marking point’s shape is used to extract the contour of the complete points correspondingly, then the contour of the complete marking point is regarded as a reference to calculate the marking point completeness.

**Figure 7:**
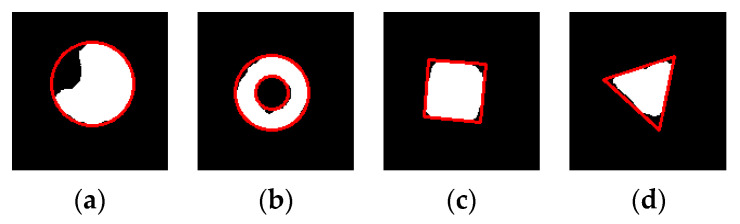
The integrity contour of various marking points: (**a**) The solid circle with integrity contour, (**b**) The hollow circle with integrity contour, (**c**) The square with integrity contour, and (**d**) The triangle with integrity contour.

For the solid circle marking point, the minimum circumscribed circle of the marking point is extracted as the contour of the complete marking point of this solid circle marking point, shown in the Figure 7a. For the hollow circle marking point, on the basis of extracting the outer circumscribed circle of the hollow circle marking point, the inner circumscribed circle is determined by the following features: the inner circle of the marking point is concentric with the outer circle. Then the contour of the complete marking point of this hollow circle marking point is extracted, shown in Figure 7b. For the square marking point, its minimum bounding square is regarded as its corresponding complete marking point contour, shown in Figure 7c. The triangle marking points are regular triangles. For the regular triangle marking point, find its minimum bounding triangle and use its longest side as a side for the regular triangle at first. Using the characteristics of the regular triangle, the contour of the complete marking point of this triangle marking point can be obtained, shown in Figure 7d.

### 4.2. Completeness Calculation of the Tire Marking Point

**Figure 8:**
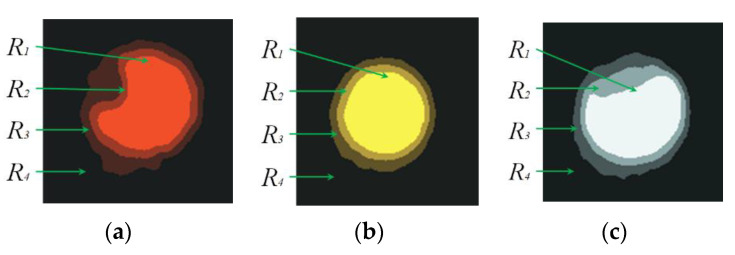
The marking point image segmentation by K-means clustering: (**a**) Segmented image for the red marking point, (**b**) Segmented image for the yellow marking point, and (**c**) Segmented image for the white marking point.

In the second part, the K-means clustering segmentation algorithm is used to segment the image into four regions *R*_1_, *R*_2_, *R*_3_, and *R*_4_, shown in Figure 8. The four regions have significant differences in the luminance channel (V channel) of the HSV space. Table 1 lists the brightness of the four regions divided by the K-means clustering for the three marking points in Figure 8. Among them, the region with the highest brightness is *R*_1_, the brightness is *t*_1_, corresponding to the object area of the marking point; the *R*_2_ and *R*_3_ regions with slightly darker brightness, the brightness is *t*_2_ and *t*_3_, respectively, corresponding to the two layers blurred edge between the object area of the marking point and the background region; the darkest region is the *R*_4_, and the brightness is *t*_4_, which corresponds to the background area of the image.

**Figure 9:**
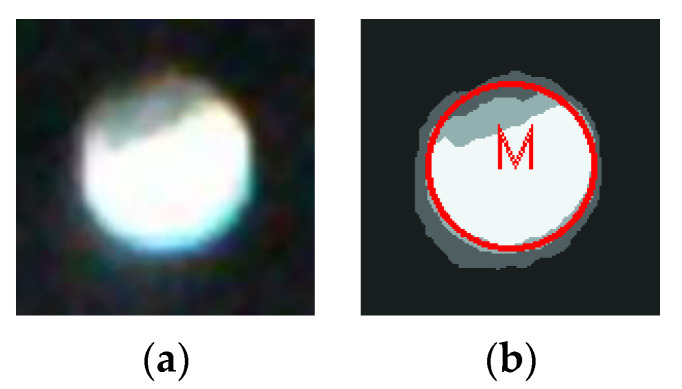
White solid circle marking point and contour extraction: (**a**) A marking point of the white solid circle, and (**b**) A contour of the complete white solid circle marking point.

By extracting region *R*_1_, the contour of the complete marking point corresponding to the object area of the marking point, the pixel points within the contour of the complete marking point are weighted and calculated, and the marking point completeness can be obtained. The method proposed in this paper not only incorporates the objective area of the marking point into the completeness calculation, but also the influence of the blurred edge on the completeness, so as to make the calculating result close to the actual marking point completeness. Taking a white solid circle marking point as an example, this method is described, shown in Figure 9a.

In Figure 9b, the red circle, named *M* is the contour of the complete marking point, and it is extracted from the solid white circle. The total number of pixels in region *M* is *M_a_*. The region *M* may contain four regions (*R*_1_*, R*_2_*, R*_3_*, R*_4_) segmented by K-means clustering. Different regions have different contributions to the marking point completeness. The region *R*_1_ is the objective of the marked point. The contribution of each pixel in the area *M* to the completeness is 1. The more the foreground pixels of the marking point in the area *M* are, the higher the marking point completeness becomes. Region *R*_4_ is the background, each background pixel in the *M* area has no contribution to the marking point completeness. The more the background pixels in the *M* area are, the lower the marking point completeness is. The *R*_2_ and *R*_3_ regions are the blurry edges between the marking point and the background. The contribution of the pixels of *R*_2_ and *R*_3_ in the *M* area for the completeness should be between 0 and 1. Since the brightness of the different regions is different, the contributions of these two areas to completeness are reflected by the brightness interpolation.

In order to accurately calculate the marking point completeness, four regions that may be contained in the *M* area are defined, and the contributions to completeness in the different regions are weighted according to their brightness:

The *R*_1_ included in the *M* region is defined as *R*_1′_*, R*_1′_ = *R*_1_ ∩ *M*, and the number of pixels is *R*_1*a*_*’*. Since *R*_1_ is the object area of the marking point, each pixel’s contribution weight *ξ*_1_ to completeness is 1.

The *R*_2_ included in the *M* region is *R*_2′_, *R*_2′_ = *R*_2_ ∩ *M*, the number of pixels is *R*_2*a*_*’*, and the calculation formula of the contribution weight *ξ*_2_ of each pixel is expressed in Equation (4):

(4) $$\xi_{2}=\left(t_{2}-t_{4}\right)\times \frac{1}{\left(t_{1}-t_{4}\right)}.$$

The *R*_3_ included in the *M* region is *R*_3′_, *R*_3′_ = *R*_3_ ∩ *M*, the number of pixels is *R*_3*a*_*’*, and the calculation formula of the contribution weight *ξ*_3_ of each pixel is expressed in Equation (5):

(5) $$\xi_{3}=\left(t_{3}-t_{4}\right)\times \frac{1}{\left(t_{1}-t_{4}\right)}.$$

The *R*_4_ included in the *M* region is *R*_4′_*, R*_4′_ = *R*_4_ ∩ *M*, the number of pixels is *R*_4*a*_*’*, and the contribution weight is *ξ*_4_ that has no contribution to its completeness.

In a word, the marking point completeness named could be calculated as Formula (6):

(6) $$D=\frac{\left(R_{1a}’\times \xi_{1}+R_{2a}’\times \xi_{2}+R_{3a}’\times \xi_{3}+R_{4a}’\times \xi_{4}\right)}{M_{a}}\times 100\%.$$

## 5. Marking Point Completeness Evaluation

The purpose of quantifying the tire marking point completeness is to determine its completeness based on the completeness rating standards given by the enterprises and shunts of the tires on the conveyor line, according to the rules of the system’s decision. The tires with unqualified marking points will be sent back to the printing module.

### 5.1. Rating Standards

According to the requirements of the enterprises, the tire marking point completeness is divided into five levels namely: intact (qualified), minor missing (qualified), medium missing (unqualified), obvious missing (unqualified), and severe missing (unqualified), the higher the degree of the marked points lack is, the lower the corresponding integrity level becomes.

For the marking points of the solid circle and hollow circle, a completeness greater than 90% is qualified, or else is unqualified, shown in Table 2.

**Figure 10:**
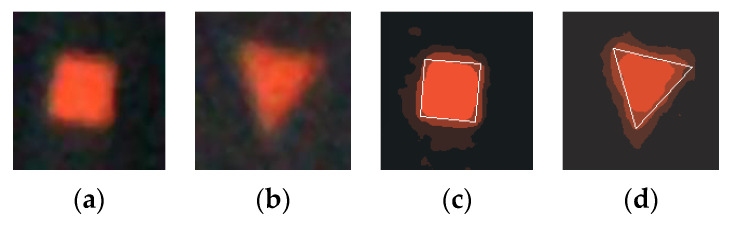
The segmenting results of square and triangle mark points: (**a**) A square marking point, (**b**) A triangle marking point, (**c**) The segmenting result of square mark point, and (**d**) The segmenting result of triangle mark point.

The square and triangle marking points have sharp corners, so the images are not clear, and the sharp corners are blurred, as shown in Figure 10a,b. The sharp corners of the marking point will be divided into the transitional edge region during the segmentation, shown in Figure 10c,d. It will lead to a calculated value of the marking point completeness lower than the solid circle and the hollow circle. So, the rating standards for the square and triangle marking points are separately formulated; that is, the rating of the squares and the triangles decreases by 4% than that in Table 2. For example, square marking points No. 20 and No. 27, and the triangle marking points No. 15 and No. 29 have been adjusted accordingly.

### 5.2. Tests and Analysis of the Marking Point Completeness

In order to examine the effectiveness of the tire marking point completeness calculation method, the manual evaluating results of the marking point completeness are used as a standard group for the comparative test. In this paper, 100 samples of the tire marking points selected to calculate the marking point completeness and use this to rate them according to the standards in Table 2. The evaluation of the marking point completeness by manual observation is subjective due to visual errors of human observation and other manual factors. In order to get objective evaluating results as far as possible, the manual rating results are obtained comprehensively by the ratings on the 100 samples from five quality inspectors of the enterprise. Due to limited space, 30 marking point images are selected and shown in Table 3. Moreover, in order to compare the K-means clustering marking point segmentation algorithm proposed in this paper with the threshold-based segmentation algorithm, the representative and advanced threshold-based algorithm, the maximum between-class variance method [15], is used and shown in Table 3. What is more, there are the results of calculating the tire marking point completeness using both the method proposed in this paper and the Otsu segmentation method in Table 3.

The Otsu algorithm and K-means clustering segmentation algorithm are used to calculate the marking point completeness. Since the segmentation methods are different, the values of the completeness calculation are also different. There are big differences of completeness values for the eleven marking points in Table 3 using these two segmentation algorithms, resulting in different ratings for marking points, including No. 3, 6, 10, 12, 15, 17, 20, 25, 27, 28, and 29 marking points. For the solid circles and hollow circle marking points, such as No. 3, 6, 10, 28, and others, due to the smearing of the marking point, when using the Otsu threshold segmentation, the marking point is slightly elliptical, so the completeness is lower than the normal one. For example, the marking point No. 28, the marking point completeness is 93.1% using the Otsu algorithm, and the corresponding rating level is 2. The marking point completeness is 96.7% using the K-means clustering segmenting algorithm, and the corresponding rating level is 1. The latter is closer to the manual rating (level 1) than the former. However, regardless of level 1 or 2, the marking point completeness is qualified. For the marking point No. 20, the marking point completeness calculated by the Otsu algorithm is 87.5%, the corresponding level is 3, which is obviously missing and is unqualified. Its completeness is 92.8% using the K-means clustering segmentation algorithm, and the corresponding rating level is 2, which is a minor missing and is a qualified marking point. The manual rating is level 2, which is qualified. In other words, the marking point No. 20 is rated as the unqualified one using the Otsu algorithm, which is inconsistent with the manual rating. The reason is that the image of the square marking point is not clear, especially at sharp corners. The marking point is divided into foreground and background by the Otsu algorithms, and the segmentation of the blurred edge area is not accurate, so the accuracy of the marking point completeness is low. This shows that in the marking point segmentation process of the foreground and background, the two segmented parts cannot reflect the influence of the different regions on the completeness. The marking point completeness calculation method of weighting based on the clustering hierarchy segmentation can not only segment the foreground of the marking point accurately, but also reflect the contribution degree of the different regions to the marking point completeness value.

Taking the manual rating as a reference, there are 28 samples correctly rated by using the method proposed in this paper among the 30 sample points. To pay attention to the marking point No. 6, which is wrongly rated, although the marking point is rated differently, the completeness calculated by the automatic recognition system is 91.5%, which belongs to the level of minor missing and it is qualified. The result of the manual rating is complete and the marking point is also qualified. The evaluation result of the automatic rating system is consistent with that of the manual rating. To pay attention to the marking point No. 26, which is wrongly rated, the completeness calculated by the automatic rating system is 90.3%, which belongs to the level of minor missing, and it is qualified. However, the result of manual ration belongs to the medium missing, and it is unqualified. The evaluation results of automatic rating system and manual rating are not consistent. Compared with the marking point No. 25, the completeness calculated by the automatic rating system is 90.5%, which belongs to the minor missing, and the marking point is qualified. The manual rating belongs to the minor missing, it is qualified. The evaluation results of the automatic rating system and the manual rating are consistent. The calculated completeness values of the marking points No. 25 and 26 are almost the same, but the manual evaluation is different. The reason is that the missing area of the marking point No. 25 is more scattered than that of the marking point No. 26, and its visual impact is weaker than that of the marking point No. 26. It can also be seen that the manual observation is difficult to quantify the completeness with a unified standard, and blurring of the boundary is inevitable. Taking the manual ratings of the enterprise quality inspection group as the standard, the completeness accuracy of the automatic recognition system is 95% for the 100 tire marking samples and its accuracy of the qualified evaluation is 99%.

In addition, the average processing speed of each marking point image is 9.27 ms for the above 100 marking point samples. There are always two marking points in each tire, and the tire marking point completeness for both of them should be calculated. The processing time for the two marking points is approximately 19 ms. The processing speed of the recognition stage for the original image is 93.7 ms per image (The marking point recognition is omitted in this paper and please read the reference [19]). Therefore, the average processing speed of each tire image in the entire marking point recognition system is 112.7 ms. It is far less than 1 s, which is required. It meets the real-time requirement of the tire marking point completeness processing.

## 6. Conclusions

A method is proposed to calculate and evaluate the tire marking point completeness based on the K-means clustering segmentation. It is as follows:

(1) The image of the marking point is enlarged to improve the accuracy of the marking point completeness; the image is segmented by the K-means clustering segmentation algorithm and divided into four regions to obtain the object area of the marking point.
(2) According to the shape of the marking point, the contour of the complete marking point is extracted; the pixels within the contour of the complete marking points are weighted based on the brightness differences of the different regions, and the marking point completeness is calculated.
(3) The marking point completeness is rated according to its calculated result. Taking the manual rating as the reference, the 100 tire marking point samples are tested. The rating accuracy of the marking point completeness is 95% and the qualified evaluation accuracy of the marking point completeness is 99%. At the same time, the average completeness calculation time of each marking point is 9.27 ms. Therefore, the method of the tire marking point completeness evaluation can meet not only the accuracy requirement but also the real-time performance of the automatic tire marking point completeness rating and the evaluation in smart factories.

## Figures and Tables

**Table 1:** The brightness of four regions after the marking point is segmented.

Segmented Region	Brightness	Red Point	Yellow Point	White Point
*R*_1_	*t*_1_	0.9412	0.9725	0.9647
*R*_2_	*t*_2_	0.6118	0.7059	0.6588
*R*_3_	*t*_3_	0.2941	0.3765	0.3451
*R*_4_	*t*_4_	0.1137	0.1176	0.1137

**Table 2:** Completeness rating standards of the solid and hollow circle marking points.

Marking Point Completeness Level	Marking Point Completeness D	Marking Point Completeness
Level 1	95% < D ≤ 100%	intact (qualified)
Level 2	90% < D ≤ 95%	minor missing (qualified)
Level 3	80% < D ≤ 90%	medium missing (unqualified)
Level 4	65% < D ≤ 80%	obvious missing (unqualified)
Level 5	D ≤ 65%	severe missing (unqualified)

**Table 3:** The results of the completeness rating for some marking points.

No.	Marking Point	Completeness Value Using Otsu	Completeness Value Using K-Means Clustering	Rating Using K-Means Clustering	Manual Rating
1	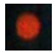	96.3%	97.0%	1	1
2	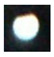	93.7%	93.8%	2	2
3	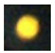	91.4%	97.8%	1	1
4	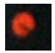	90.3%	89.8%	3	3
5	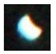	74.6%	73.6%	4	4
6	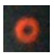	89.8%	91.5%	**2**	**1**
7	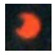	80.2%	83.1%	3	3
8	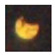	72.9%	75.6%	4	4
9	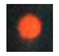	99.1%	99.0%	1	1
10	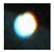	85.1%	93.6%	2	2
11	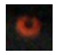	75.3%	78.6%	4	4
12	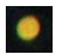	89.5%	94.3%	2	2
13	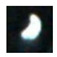	61.3%	61.7%	5	5
14	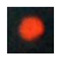	91.8%	92.9%	2	2
15	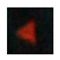	83.5%	91.0%	1	1
16	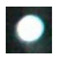	96.1%	97.8%	1	1
17	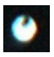	90.5%	89.7%	3	3
18	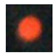	96.1%	95.7%	1	1
19	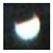	74.1%	77.8%	4	4
20	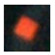	87.5%	92.8%	1	1
21	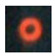	94.7%	96.4%	1	1
22	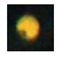	83.5%	87.9%	3	3
23	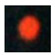	92.9%	94.0%	2	2
24	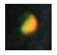	64.9%	62.9%	5	5
25	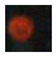	88.2%	90.5%	2	2
26	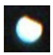	91.3%	90.3%	**2**	**3**
27	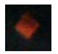	70.5%	80.1%	3	3
28	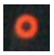	93.1%	96.7%	1	1
29	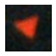	85.6%	93.1%	1	1
30	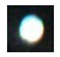	95.6%	97.5%	1	1
